# Thrombocytopenia as a Predictor of Severe Acute Kidney Injury in Patients with Hantaan Virus Infections

**DOI:** 10.1371/journal.pone.0053236

**Published:** 2013-01-02

**Authors:** Meiliang Wang, Jiuping Wang, Tianping Wang, Jing Li, Ling Hui, Xiaoqin Ha

**Affiliations:** 1 Center for Experimental Medicine, Lanzhou General Hospital, Lanzhou Military Command, Lanzhou, Gansu, China; 2 Department of Infectious Diseases, Tangdu Hospital, the Fourth Military Medical University, Xi’an, Shaanxi, China; 3 Gansu Key Laboratory of Stem Cell and Gene Therapy, Lanzhou, Gansu, China; University of Alabama at Birmingham, United States of America

## Abstract

**Background:**

Hematological abnormalities often occur several days before kidney injury in patients with hemorrhagic fever with renal syndrome (HFRS). We aimed to investigate the prevalence and prognostic value of the early hematological markers in patients with HFRS caused by Hantaan virus (HTNV) infection.

**Methods:**

In a retrospective cohort study, we analyzed the case records of 112 patients with acute HTNV infection and evaluated the hematological markers for early prediction and risk stratification of HFRS patients with acute kidney injury (AKI).

**Results:**

Of 112 patients analyzed, 66 (59%) developed severe AKI, defined as either receipt of acute dialysis or increased serum creatinine ≥354 µmol/L. The prognostic accuracy of hematological markers, as quantified by the area under the receiver-operating-characteristic curve (AUC), was highest with the nadir platelet count (AUC, 0.89; 95% CI, 0.83–0.95), as compared with the admission platelet count (AUC, 0.84; 95% CI, 0.77–0.92), and the admission and peak leukocyte counts. The nadir platelet count correlated moderately with the levels of peak blood urea nitrogen (*r* = –0.616) and serum creatinine (*r* = –0.589), the length of hospital stay (*r* = –0.599), and the number of dialysis sessions that each patient received during hospital stay (*r* = –0.625). By multivariate analysis, decreased nadir platelet count remained independently associated with the development of severe AKI (odds ratio, 27.57; 95% CI, 6.96–109.16; *P*<0.0001).

**Conclusions:**

Thrombocytopenia, rather than leukocytosis, is independently associated with subsequent severe AKI among patients with acute HTNV infection.

## Introduction

Hemorrhagic fever with renal syndrome (HFRS) is a combination of fever, hemorrhage, thrombocytopenia and acute kidney injury (AKI), among which severe AKI is the leading cause of death in patients with HFRS, particularly in the late stage. The disease is mainly caused by four closely related hantaviruses, Hantaan (HTNV) in Asia, Puumala (PUUV) and Dobrava in Europe, and Seoul virus worldwide. HTNV is the prototypic representative of this genus responsible for most cases of hantavirus infections in Asia. Unlike other hantaviruses, HTNV infections usually result in a severe form of HFRS, but the illness can range from a mild illness without oliguria, to an extremely severe illness requiring hemodialysis.

Currently, there is as yet no vaccine or specific antiviral therapy for HFRS. The treatment for patients with hantavirus infections is supportive care, including intermittent hemodialysis (IHD) and continuous *renal* replacement therapy (CRRT). Expected prognosis whether or when dialysis is started is of great importance to patients with severe HFRS and their clinicians, and for planning of treatment guidelines. Most of the symptoms and signs currently used in classifying HFRS [1], [2], such as oliguria, anuria and kidney injury, do not appear until the later stages of illness. Thrombocytopenia is an early, consistent process during hantavirus infection, and is a major diagnostic feature in patients with HFRS [3]. In a cohort of patients infected with PUUV, which causes a mild form of HFRS in Europe, previous study showed that low platelet count (<60×10^9^/L) was significantly associated with the subsequent severe AKI [4]. This study used the platelet count obtained at the initial evaluation (1–9 days after symptom onset) to classify thrombocytopenia that may still be normal or have already returned to normal in some patients. Other attempts have provided a list of symptoms, signs, and hematological, biochemical or immunological parameters, that could be associated with severe HFRS [5]–[9], but how these parameters should be applied for clinical diagnosis is not apparent. To date, no prognostic models are available for patients with HTNV infection in Asia.

Acute hantavirus infection is a highly dynamic process, characterized by a short transient thrombocytopenia followed by mild-to-severe AKI [4]. We therefore assessed the extent to which the early hematological abnormalities, such as thrombocytopenia and leukocytosis, predicted the later biochemical abnormalities, such as the increases in levels of blood urea nitrogen and serum creatinine reflecting the severity of AKI, in patients with HTNV infection.

## Methods

### Study Population

We retrospectively reviewed the case records of 125 patients with HFRS, diagnosed during the major HTNV epidemic periods, from October through December, in 2008 and 2009, at the Tangdu Hospital of the Fourth Military Medical University in Xi’an. The clinical diagnosis of acute HTNV infection was serologically confirmed by an IgM-capture ELISA (Lanzhou Institute of Biological Products, China) according to the manufacturer’s instructions for the detection of virus-specific IgM antibody. The levels of IgM antibodies were scored as follows: 0, negative; 1+, mildly positive; 2+, moderately positive; and 3+, strongly positive. Patients were included if they had a final serological score of 1+ or greater. Exclusion criteria included acute dialysis requirement within 24 h of admission. The study was approved by the ethics committees of the Lanzhou General Hospital and the Fourth Military Medical University. Informed consent was not required as it was a retrospective study and the data were analyzed anonymously. Both ethics committees specifically waived the need for consent.

### Clinical Data Collection

Clinical and laboratory data were obtained daily throughout hospitalization and were collected on standardized data collection forms. Data requested from participating patients included demographic information, platelet count, leukocyte count, hematocrit, blood urea nitrogen, serum creatinine, uric acid, albumin, aspartate aminotransferase, alanine aminotransferase, the length of hospital stay, the need for hemodialysis treatment, the number of dialysis sessions, and the presence of shock, proteinuria, hematuria, and severe complications. All subjects were admitted to the hospital and monitored daily until discharged. IHD or CRRT treatment was guided by the ward physician based upon clinical necessity.

### Statistical Analysis

Continuous variables were presented as medians with the interquartile range (IQR), and categorical variables as numbers and percentages. Continuous variables were compared with the use of the nonparametric Mann-Whitney *U* test and categorical variables with the use of the Pearson’s χ^2^ test or Fisher’s exact test when appropriate. Spearman correlations and linear regression analyses were used to evaluate the relations between the early hematological parameters and the later biochemical, hematological, or clinical parameters. Receiver-operating-characteristic (ROC) curves were constructed to assess the sensitivity and specificity of the platelet and leukocyte counts in predicting the development of severe AKI and the need of dialysis. The comparison of areas under the ROC curves (AUC) was performed as recommended by DeLong et al. Multivariate logistic regression model was used to identify hematological factors associated with the development of severe AKI. Covariates entered into the model included age, gender, and the presence of shock, proteinuria and hematuria preceding the development of severe AKI. All *P* values are two-sided, and *P* values of <0.05 were considered significant. Analyses were done with PASW statistics, version 18.0 for windows (SPSS), and MedCalc software, version 11.5.0.0 (MedCalc).

## Results

### Characteristics of the Patients

In total, 125 patients were enrolled. Of these patients, 112 (90%) were eligible to be included in the study. The reasons for ineligibility were: patient had an undetermined diagnosis due to the lack of serological HTNV antibodies (n = 2); and patient received acute dialysis on admission due to severe AKI (n = 11).

Patients with acute HTNV infections, 14 females and 98 males, were admitted to the hospital 5 days (IQR 4–6 days) after acute onset of clinical symptoms. The most common laboratory findings were thrombocytopenia, leukocytosis and AKI. Thrombocytopenia (nadir platelet count <100×10^9^/L) occurred in 110 (98%) patients, and severe thrombocytopenia (≤33×10^9^/L) in 71 (63%) patients. Leukocytosis (peak leukocyte count >10×10^9^/L) was recorded in 102 (91%) patients, and severe leukocytosis in 54 (48%) patients with a leukocyte count of 18.7×10^9^/L or more. Moreover, the increases in levels of serum creatinine (>133 µmol/L) were noted in 101 (90%) patients, 66 (65%) of which subsequently developed severe AKI, defined as receipt of acute dialysis or increased serum creatinine ≥354 µmol/L by Acute Kidney Injury Network (AKIN) criteria for stage 3. Fifty-seven patients (51%) received acute dialysis and 2 (2%) died. Patients developing severe AKI were older, and more likely to have shock, hematuria, thrombocytopenia and leukocytosis, need acute dialysis, and therefore stay longer in hospital than patients who did not develop severe AKI (Table 1).

**Table 1:** Characteristics of patients by severe AKI status.

	Severe AKI (n = 66)	No severe AKI (n = 46)	*P* value
Demographic characteristics			
Age (yr)	44 (33–56)	34 (24–45)	0.002
Male sex	57 (86%)	41 (89%)	0.663
Hematological markers			
Admission platelet count (×10^9^/L)	24 (11–31)	51 (36–73)	<0.0001
Nadir platelet count (×10^9^/L)	14 (9–25)	45 (34–68)	<0.0001
Admission leukocyte count (×10^9^/L)	17.9 (11.5–25.1)	11.5 (8.0–15.9)	0.001
Peak leukocyte count (×10^9^/L)	21.3 (15.3–31.4)	15.5 (10.4–21.2)	0.0001
Nadir hematocrit (%)	29.5 (24.4–32.8)	36.9 (35.0–38.9)	<0.0001
Renal function			
Peak blood urea nitrogen (mmol/L)	30.2 (25.9–35.4)	11.3 (7.6–16.1)	<0.0001
Peak serum creatinine (µmol/L)	729 (539–956)	181 (132–240)	<0.0001
Peak uric acid (µmol/L)	511 (392–630)	549 (467–659)	0.185
Clinical records			
Shock	16 (24%)	4 (9%)	0.035
Proteinuria	60 (91%)	41 (89%)	0.758
Hematuria	47 (71%)	10 (22%)	<0.0001
Dialysis required	57 (86%)	0 (0%)	<0.0001
Length of hospital stay (days)	21 (14–29)	9 (7–11)	<0.0001

Data are median (IQR) or number (%).

### Association of Hematological Markers with Renal Function

Thrombocytopenia is an early, common and consistent laboratory finding in patients with acute HTNV infection. The early decreasing platelet counts reached their nadirs 6 days (IQR, 5–7 days) after the acute onset of fever, followed by consistent rises in levels of blood urea nitrogen and serum creatinine in patients developing severe AKI reaching the maximums 8 days (IQR, 7–11 days), and 11 days (IQR, 8–16 days), respectively. In these patients, platelet count nadirs preceded the peaks of blood urea nitrogen and serum creatinine by 2 days (1–5 days) and 5 days (3–9 days), respectively. Ten days (IQR, 9–11 days) after the onset of symptoms, platelet counts returned to normal in all patients, with values >100×10^9^/L. Leukocytosis, reflecting the inflammatory process during hantavirus infection, peaked almost at the same time as thrombocytopenia (median, 7 days; IQR, 5–8 days), but may occur or reoccur at the later stages of the disease.

**Figure 1:**
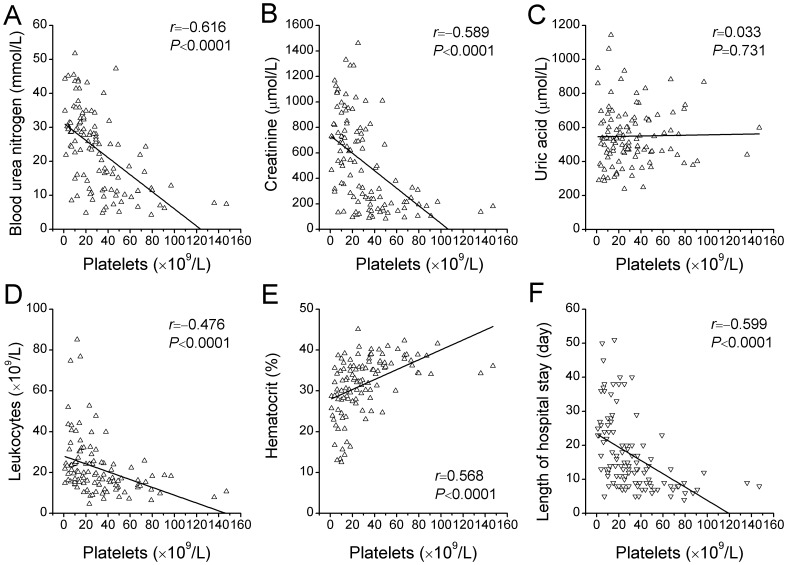
Correlations of nadir platelet count with other laboratory or clinical findings. Shown are the levels of peak blood urea nitrogen (*A*), peak serum creatinine (*B*), peak uric acid (*C*), peak leukocyte count (*D*) and nadir hematocrit (*E*), and lengths of hospital stay (*F*) plotted against the nadir platelet count. The *r* denotes the Spearman correlation coefficient, and the line the linear regression for each comparison.

The nadir platelet count correlated inversely with the peak blood urea nitrogen (*r* = –0.616, *P*<0.0001), peak serum creatinine (*r* = –0.589, *P*<0.0001), peak leukocyte count (*r* = –0.476, *P*<0.0001) and the length of hospital stay (*r* = –0.599, *P*<0.0001), and correlated positively with the nadir hematocrit (*r* = 0.568, *P*<0.0001), but not with the peak uric acid (*r* = 0.033, *P* = 0.731), though hyperuricemia is very common in patients with HFRS (Figure 1).

Significant but weaker correlations were found, moreover, between the peak leukocyte count and the peak blood urea nitrogen (*r* = 0.344, *P* = 0.0002), peak serum creatinine (*r* = 0.432, *P*<0.0001), nadir hematocrit (*r* = –0.287, *P* = 0.002) and the length of hospital stay (*r* = 0.348, *P* = 0.0002).

### Association of Hematological Markers with AKI Development

**Figure 2:**
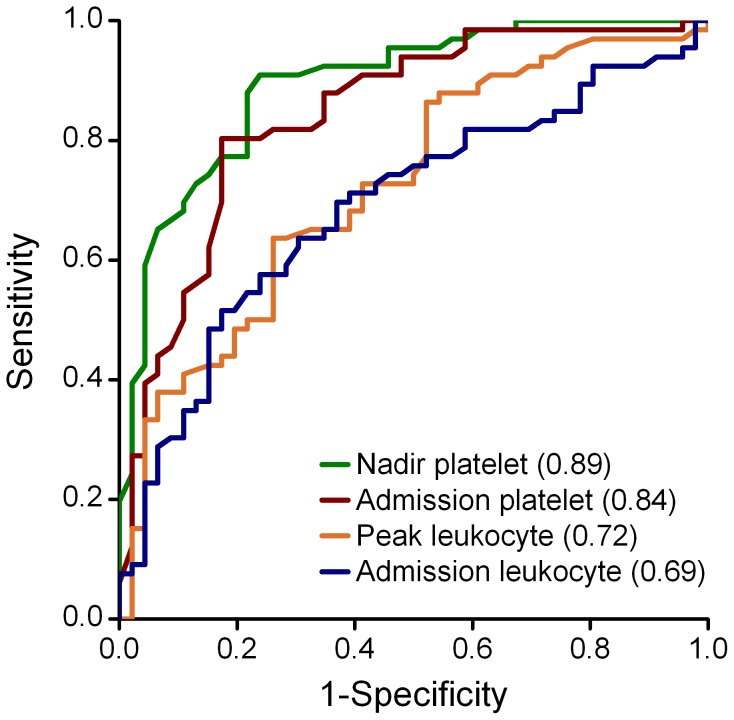
Receiver-operating-characteristic (ROC) curves for hematological markers to predict the development of severe AKI. Severe AKI is defined as the receipt of acute dialysis or increased serum creatinine ≥354 µmol/L.

The admission and nadir platelet counts were significantly lower in patients in whom severe AKI subsequently developed than in those without severe AKI (Table 1). In contrast, the admission and peak leukocyte counts were significantly higher in patients who had subsequent severe AKI than in those without. The accuracy for the prediction of severe AKI, as quantified by the AUC, was significantly higher with the nadir platelet count than that with the admission platelet count (AUC for nadir platelet, 0.89; 95% CI, 0.83–0.95; vs. AUC for admission platelet, 0.84; 95% CI, 0.77–0.92; *P* = 0.028) and was also significantly higher than that with the admission and peak leukocyte counts (AUC for admission leukocyte, 0.69; 95% CI, 0.59–0.79; and for peak leukocyte, 0.72; 95% CI, 0.63–0.82; *P*<0.0001 and *P* = 0.0003, respectively) (Figure 2). At a cut-off value of 33×10^9^/L, the sensitivity and specificity to predict severe AKI were 91% and 76% for nadir platelet count, respectively (Table 2). After adjustment for age, gender, peak leukocyte count and for other variables preceding the development of AKI, decreased nadir platelet count (odds ratio, 27.57; 95% CI, 6.96–109.16; *P*<0.0001), and the presence of hematuria (odds ratio, 8.99; 95% CI, 2.32–34.81; *P* = 0.001) remained independent predictors of severe AKI (Table 3).

**Table 2:** Sensitivity, specificity, and predictive values for the development of severe AKI using hematological cut-off values.

	Cut-off value	Sensitivity	Specificity	PPV	NPV
Admission platelet (×10^9^/L)	17	0.39	0.96	0.93	0.52
	34	0.80	0.83	0.87	0.75
	62	0.98	0.41	0.71	0.95
Nadir platelet (×10^9^/L)	17	0.59	0.96	0.95	0.62
	33	0.91	0.76	0.85	0.85
	49	0.98	0.39	0.70	0.95
Admission leukocyte (×10^9^/L)	17.3	0.52	0.83	0.81	0.54
Peak leukocyte (×10^9^/L)	18.7	0.64	0.74	0.78	0.59

PPV, positive predictive value; NPV, negative predictive value.

**Table 3:** Multivariable logistic regression for the prediction of severe AKI combining nadir platelet count with other variables.

	Adjusted odds ratio	95% CI	*P* value
Age (per year)	1.04	1.00–1.08	0.071
Gender	0.92	0.16–5.32	0.925
Presence of shock	1.88	0.28–12.64	0.516
Presence of proteinuria	1.09	0.17–7.11	0.932
Presence of hematuria	8.99	2.32–34.81	0.001
Nadir platelet ≤33×10^9^/L	27.57	6.96–109.16	<0.0001
Peak leukocyte ≥18.7×10^9^/L	1.16	0.33–4.05	0.821

### Association of Thrombocytopenia with Dialysis Treatment

**Figure 3:**
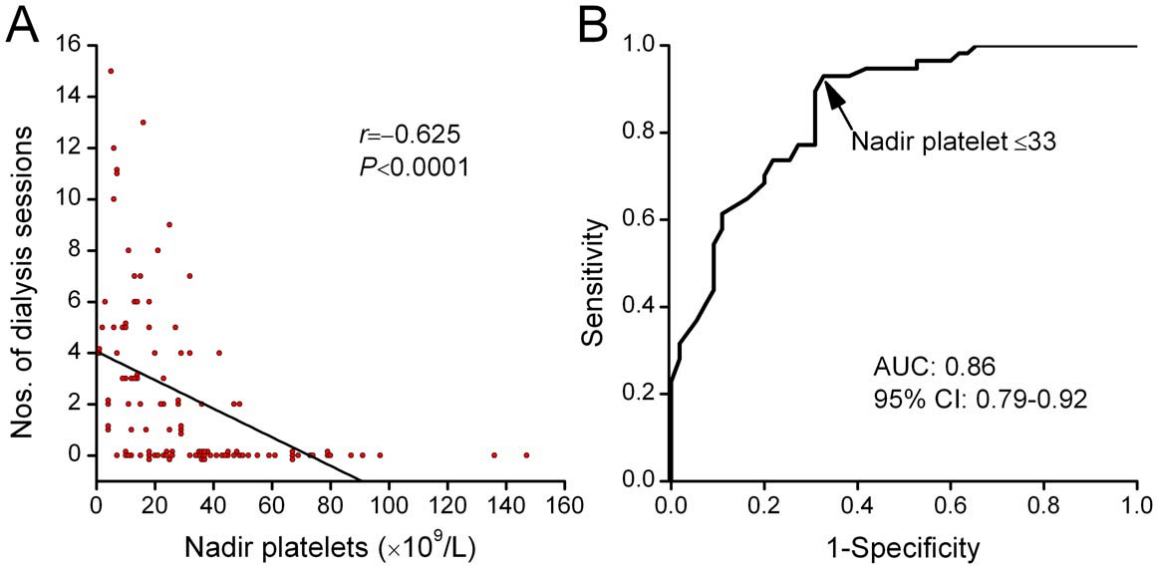
Nadir platelet count associated with dialysis. (A) Nadir platelet count correlated with the number of dialysis sessions each patient received during hospitalization. (B) Shown are receiver-operating-characteristic (ROC) curves for nadir platelet count in predicting the need of dialysis.

Of 112 patients, 57 (51%) received one or more sessions of IHD and/or CRRT treatment. We observed a moderate correlation between the nadir platelet count and the number of dialysis sessions that each patient received during hospital stay (*r* = –0.625, *P*<0.0001) (Figure 3A). The nadir platelet counts were significantly lower in patients receiving acute dialysis compared with those not receiving dialysis (median, 14×10^9^/L vs. 39×10^9^/L; *P*<0.0001 ), but there were no differences between nadir platelet counts in patients who received one *versus* more than one dialysis session. The AUC for nadir platelet count in the prediction of dialysis was 0.86 (95% CI, 0.79–0.92) (Figure 3B). At a cut-off level of 33×10^9^/L, the sensitivity and specificity of nadir platelet count in predicting dialysis were 93% and 67%, respectively.

### Association of Thrombocytopenia with Adverse Outcomes

Patients who had thrombocytopenia during the early stage of infection had more frequently experienced adverse outcomes. Of 71 patients with nadir platelet count ≤33×10^9^/L, 7 (10%) developed pulmonary complications including pulmonary *infection,* edema, *hemorrhage, and* respiratory distress or failure, 4 (6%) experienced severe brain complications including *cerebral* edema, hemorrhage, and uremic encephalopathy, 6 (8%) developed acute pancreatitis, 1 (1%) had multiorgan failure, 10 (14%) received one or more times of urgent rescue medications, and 2 (3%) died. *In contrast, none of 41 patients who had* a nadir platelet count of more than 33×10^9^/L developed these complications.

## Discussion

In this study, the clinical processes of patients with acute HTNV infection were retrospectively followed throughout hospitalization. During the course of acute HTNV infection, the patients sequentially developed mild-to-severe thrombocytopenia, leukocytosis, and AKI. In these patients, the nadir platelet count negatively correlated with the levels of blood urea nitrogen and serum creatinine, the number of dialysis sessions, and the length of hospital stay reflecting the severity of renal dysfunction. Severe thrombocytopenia in patients with acute HTNV infection, independent of leukocytosis, was strongly associated with a substantially increased risk for development of severe AKI.

Little is known about the prognostic importance of thrombocytopenia in patients with acute hantavirus infections. Approximately half of the patients (7 of 15 or 47% of patients) in the study of PUUV infection had severe thrombocytopenia [4]. These patients also had a higher level of serum creatinine and a higher prevalence of severe AKI. In these patients, thrombocytopenia was defined as an initial platelet count <60×10^9^/L, a much higher threshold than that used in our cohort of patients who had a much more severe form of HFRS caused by HTNV infection.

By contrast with this study [4], the platelet count was also measured in one other study of acute HTNV infection in which 24 (39%) of 61 patients had thrombocytopenia, defined as admission platelet count ≤38×10^9^/L [6]. The admission leukocyte count, rather than the admission platelet count, was found to be significantly associated with the development of oliguric AKI, which was defined as a urine output <400 mL/24 h (the significant difference of the minimum platelet count between patients with and without AKI was not described further). Similarly, a recent study in a cohort of 36 PUUV-infected patients showed that the maximum leukocyte count, but not the minimum platelet count, was a weak risk factor for severe AKI [10]. The details about the clinical courses of the patients, however, were not available for measuring the discrepancy.

Although most of these studies used a threshold of initial platelet count to define thrombocytopenia, nadir platelet count does reflect the degree of thrombocytopenia more sensitively. Our study shows that in HTNV cohort, up to 71 of 112 (63%) had a nadir platelet count of 33×10^9^/L or less. The incidence of thrombocytopenia was higher than previously reported both in the PUUV and HTNV cohorts [4], [6].

In our study, the severities of thrombocytopenia and leukocytosis were comparable to those in the previous HTNV study by Kim et al [6], but were more severe than those in the PUUV studies [4], [10]. The nadir platelet count, as well as the peak leukocyte count, was found to be correlated with the laboratory and clinical parameters reflecting the severity of the disease (Figure 1). The diagnostic accuracy for severe AKI was highest with the nadir platelet count, as compared with the admission platelet count, and the admission and peak leukocyte counts (Figure 2). Similar to the previous HTNV study [6], the presence of hematuria preceding the development of severe AKI was also an independent predictor (Table 3). We further observed that the nadir platelet count was moderately correlated with the number of dialysis sessions each patient received during hospital stay and ROC curve yielded an AUC of 0.86 for predicting dialysis (Figure 3).

Thrombocytopenia is a consistent finding among viral hemorrhagic fever [11]. Several mechanisms *may be postulated to* explain the cause of thrombocytopenia associated with these diseases, including impaired proplatelet formation and platelet release [12], *coagulation abnormalities* [13], [14] and disseminated intravascular coagulation that correlated with a more severe disease [15]. Similar to HFRS, thrombocytopenia has also been shown to be associated with the development of complications in dengue fever [16], [17], and a platelet count of 50 ×10^9^/L or less on days 5 to 7 of illness has been used as a marker of severe disease [18]. Similarly, a significantly lower platelet count was observed in fatal cases than in non-fatal cases with Crimean-Congo hemorrhagic fever [19], [20], and a platelet count of ≤20×10^9^/L was independently associated with high mortality [14], [20]. However, the mechanism underlying thrombocytopenia in patients with severe diseases is not known.

Among the patients with severe AKI, timely initiation of dialysis may reduce subsequent complications and death risk [21]. The platelet count may make it possible both to rule in and to rule out the prognosis of severe AKI and need of dialysis on the basis of the daily measurement. Despite the good performance of the platelet category in the early diagnosis of severe AKI caused by HTNV infection, it should be used only in combination with the initial clinical manifestations of HFRS, such as fever and hemorrhage.

One limitation of this study is its small cohort, therefore, these markers require validation in independent cohorts. Another limitation is that this study can not differentiate the peak levels of blood urea nitrogen and serum creatinine between patients with and without IHD or CRRT treatment. The values were distinctly underestimated in patients who had undergone dialysis; the coefficient was thereby underascertained. Other early abnormalities, such as elevated hematocrit, hypoalbuminemia, and elevated aspartate aminotransferase and alanine aminotransferase indicating the presence of hemoconcentration and liver impairment, were also noted in acute HTNV infection, but were not included in the analysis because they are not measured routinely in patients with HFRS.

In summary, early thrombocytopenia was associated with the development of severe AKI, need of dialysis, and length of hospital stay. Because platelet count is a simple, readily available clinical test that is widely used in medical care, and pronounced thrombocytopenia is an early and consistent laboratory abnormality in HFRS, it might be of value in risk stratification of patients at high risk of developing severe complications for the timely initiation of hemodialysis treatment. Of potential interest to physicians is whether therapeutic increase in platelet count might be useful in the prediction of improvement in clinical severities.
